# Modeling of Self-Assembled Peptide Nanotubes and Determination of Their Chirality Sign Based on Dipole Moment Calculations

**DOI:** 10.3390/nano11092415

**Published:** 2021-09-16

**Authors:** Vladimir Bystrov, Alla Sidorova, Aleksey Lutsenko, Denis Shpigun, Ekaterina Malyshko, Alla Nuraeva, Pavel Zelenovskiy, Svitlana Kopyl, Andrei Kholkin

**Affiliations:** 1 Institute of Mathematical Problems of Biology, The Branch of Keldysh Institute of Applied Mathematics, RAS, 142290 Pushchino, Russia; 2 Faculty of Physics, Lomonosov Moscow State University, 119991 Moscow, Russia; sky314bone@mail.ru (A.S.); aleksluchrus@yandex.ru (A.L.); denish.den@mail.ru (D.S.); katyamalyshko@mail.ru (E.M.); 3 School of Natural Sciences and Mathematics, Ural Federal University, 620000 Ekaterinburg, Russia; allanuraeva@gmail.com (A.N.); zelenovskiy@urfu.ru (P.Z.); kholkin@ua.pt (A.K.); 4 Department of Physics & CICECO-Aveiro Institute of Materials, University of Aveiro, 3810-193 Aveiro, Portugal; svitlanakopyl@ua.pt; 5 Physical Materials Science and Composite Materials Centre, Research School of Chemistry & Applied Biomedical Sciences, National Research Tomsk Polytechnic University, 634050 Tomsk, Russia

**Keywords:** dipeptides, diphenylalanine, helical structures, peptide nanotubes, self-assembly, molecular modeling, dipole moments, polarization, chirality

## Abstract

The chirality quantification is of great importance in structural biology, where the differences in proteins twisting can provide essentially different physiological effects. However, this aspect of the chirality is still poorly studied for helix-like supramolecular structures. In this work, a method for chirality quantification based on the calculation of scalar triple products of dipole moments is suggested. As a model structure, self-assembled nanotubes of diphenylalanine (FF) made of L- and D-enantiomers were considered. The dipole moments of FF molecules were calculated using semi-empirical quantum-chemical method PM3 and the Amber force field method. The obtained results do not depend on the used simulation and calculation method, and show that the D-FF nanotubes are twisted tighter than L-FF. Moreover, the type of chirality of the helix-like nanotube is opposite to that of the initial individual molecule that is in line with the chirality alternation rule general for different levels of hierarchical organization of molecular systems. The proposed method can be applied to study other helix-like supramolecular structures.

## 1. Introduction

Self-assembly of biomolecules such as amino acids, nucleotide bases, phospholipids, and oligo- and polypeptides is the basis for the formation of DNA, molecular motors, viruses, and many other biological systems [1,2,3,4]. Biomimetic self-assembly is also a promising bottom-up approach for nanomaterials design in nanobiotechnology [5,6]. Helical self-organizing structures of different levels of hierarchical organization is an often result of such self-assembly [1,4,5,7,8]. Among them, α-helices, a common type of regular secondary structure of many proteins, are the simplest and most energetically favorable structures [1,6]. For natural proteins consisting of L-amino acids, a chirality sign alternation from the “left-handed” type to the “right-handed” is observed at different levels of hierarchical organization [9,10,11,12]. In the case of DNA, the sequence of the chirality sign is “right-handed deoxyribose carbohydrate”–“left-handed nucleotides”–“right-handed DNA double helix”–“left-handed superhelix” with the increasing complexity of their level of organization. This feature of chirality is one of the key points in the hierarchy and self-organization of any biological system [9,10,11,12].

Despite the concept of chirality, in its qualitative sense, being widespread in natural sciences, its quantitative aspects (the magnitude and sign of chirality) are still poorly studied [13,14,15,16,17,18,19,20,21,22,23,24]. In structural biology, it is of great importance to obtain the quantitative estimates of the magnitude of chirality and the chirality sign to compare both molecular constructs with the same symmetry type and those with different types of the symmetry. The problem still lacks a solution, although many studies have been performed in the field. The detailed analysis of these studies can be found in [23].

Recently, a new method for the chirality quantification based on the scalar triple product of three consecutive vectors connecting Cα carbon atoms of neighboring amino acid residues in the polypeptide helical or superhelical structures was proposed [21,22,23,24]. Despite this approach being successfully applied to a variety of proteins [24] taken from the Protein Data Bank [25], until now it has not been used for helix-like supramolecular structures such as peptide nanotubes.

Peptide nanotubes (PNTs) is an important example of helix-like self-organizing supramolecular systems [5,26,27]. Peptides and their derivatives were recognized recently as biological and bio-inspired building blocks for the construction of various advanced functional materials for nanotechnology and biomedicine [28,29]. Short, linear peptides containing aromatic amino acid residues such as phenylalanine (F, H-Phe-OH) attract special attention due to their ability to mimic the self-organizing behavior of more complex proteins [26].

**Figure 1:**
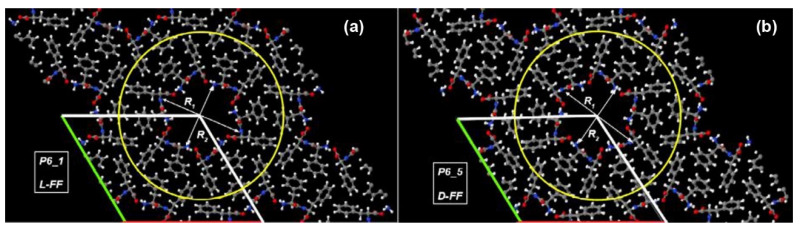
Images of molecular crystals composed of: (**a**) L-FF PNTs (space group P6_1_), and (**b**) D-FF PNTs (space group P6_5_). Hexagonal unit cells are marked with green, red, and white lines. The individual PNTs in crystal are highlighted by yellow circles. Atom colors: oxygen—red, nitrogen—blue, carbon—grey, and hydrogen—white.

The simplest aromatic dipeptide capable to form helix-like nanotubes is diphenylalanine (FF, H-Phe-Phe-OH) [30,31,32,33,34,35,36,37,38,39,40]. Each turn of such helix PNT consists of six FF molecules (Figure 1). Fast self-assembly of such PNTs occurs in aqueous media, and the variation of external conditions allows tuning the PNT’s growth rate, length, and their physical properties [33,38,39,41,42,43,44,45]. FF PNTs possess a wide range of useful functional properties [41,46,47,48] that make them promising material for various applications in nanotechnology [33,41,46,47,48,49,50], nanoelectronics [28,35,48,51,52], and biomedicine [34,35,36,47,50,53,54,55].

The effect of chirality of FF molecules on the self-assembly and properties of PNTs has recently been studied in detail both experimentally and by computer simulation [37,38,42,56,57,58,59]. Lattice cell parameters for PNT made of “right-handed” FF molecules (H-D-Phe-D-Phe-OH, abbreviated hereafter D-FF) are close to those for PNTs made of “left-handed” FF molecules (H-L-Phe-L-Phe-OH, abbreviated hereafter L-FF), but their space groups are different [38]. Due to the difference of FF monomers chirality, the L-FF PNTs belong to the P6_1_ space group, whereas D-FF PNTs belong to the P6_5_. This P6_1_–P6_5_ pair is one of 11 pairs of enantiomorphic space groups [60] that are distinguished by the twisting direction of their 6-fold screw axis [61]. It was also shown that “left-handed” L-FF molecules form “right-handed” helix-like PNTs, whereas “right-handed” D-FF molecules form “left-handed” PNTs distinguished with their intermolecular interaction energies, self-assembly kinetics, and characteristic lengths [30,38].

In this work, we demonstrated that the method for chirality quantification proposed recently for protein helical structures [23,24] can be adopted for analysis of helix-like self-assembled PNTs. For the calculation of magnitude and sign of the chirality of L-FF and D-FF PNTs a set of sequential vectors of individual dipole moments of FF molecules comprising the turn of each helix of PNTs was used. The dipole moments were calculated using the HyperChem software [62].

## 2. Models Details and Computational Methods

### 2.1. Main Models and Used Software

Recently, we studied the structure and properties of empty L-FF and D-FF PNTs, as well as those with inner cavity filled with water molecules [56,57]. As water molecules do not affect the chirality of the PNTs, in this work, we considered empty (anhydrous) PNTs to simplify the calculations. The initial models of the PNTs were constructed using the same approach as in [56,57] based on X-ray crystallographic data for L-FF PNT (CCDC 16337, work [31]) and for D-FF PNT (CCDC 1853771, work [38]) taken from Cambridge Crystallographic Data Center (CCDC) [63]. The structural optimization and calculations were carried out using the density functional theory (DFT) methods (in Vienna Ab initio Simulation Package (VASP) program [64]), taking into account the Van der Waals interactions by “PBE + D3” method. The resulted molecular structures visualized by CCDC Mercury [65] are presented in Figure 1, whereas their main crystallographic parameters are summarized in Table 1.

### 2.2. Models of FF Nanotubes

**Figure 2:**
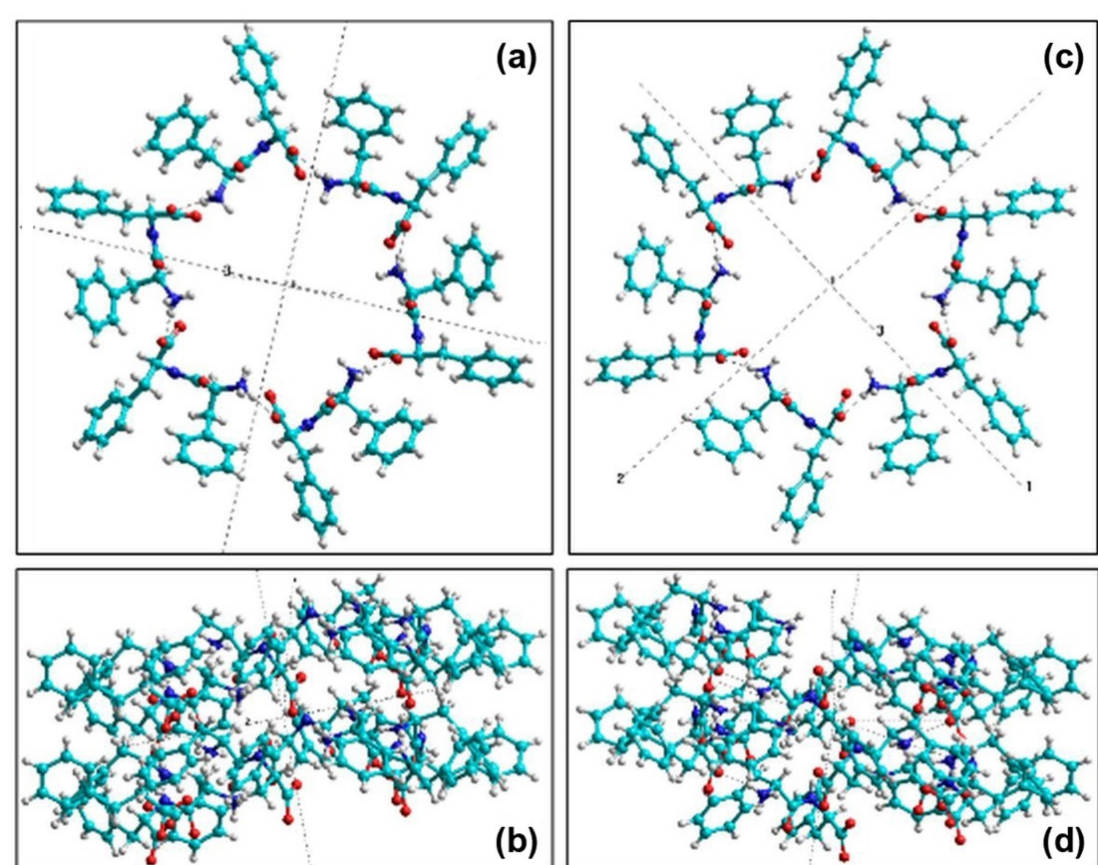
Two coils of FF PNT in HyperChem workspace based on experimental crystallographic data [31,38]: (**a**) L-FF in the Z-plane, (**b**) L-FF in the Y-plane, (**c**) D-FF in the Z-plane, and (**d**) D-FF in the Y-plane.

**Figure 3:**
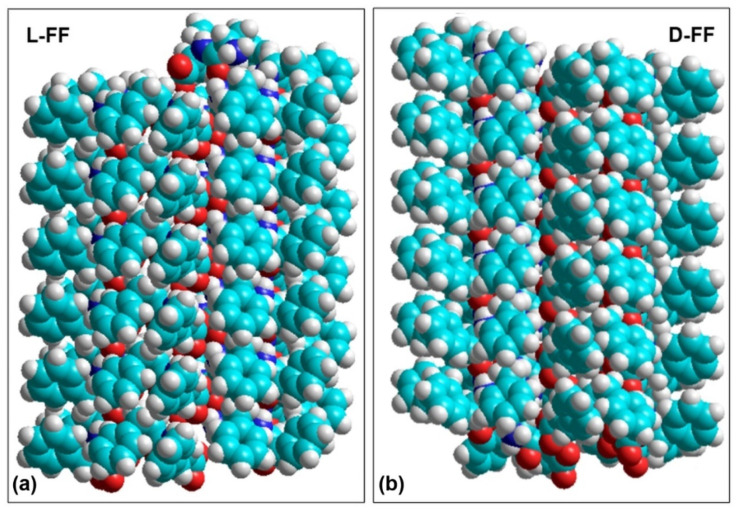
Images of helix-like FF PNTs based on experimental crystallographic data [31,38]: (**a**) L-FF and (**b**) D-FF. L-FF PNT shows right-handed twist, whereas D-FF shows left-handed.

The molecular structures of both L-FF and D-FF PNTs were converted using OpenBabel software [66] from *.cif to *.hin format for their further analysis and calculations of their polar properties with various molecular mechanical and quantum-mechanical semi-empirical methods in HyperChem package [62] (Figure 2). These structures contain two coils of the helix arranged along with the *c* axis. Each coil consists of 6 FF molecules (258 atoms) and coils are separated with a lattice constant *c* around 5.45 Å [56]. The repetition of the coils along the c-axis leads to the formation of PNT with the corresponding chirality: right-handed helix for L-FF and left-handed helix for D-FF (Figure 3).

It is known that water molecules in PNT nanochannels can affect both the structure and properties of the PNTs [41,44,56,57]. Therefore, in this work we considered in more detail the empty (anhydrous) nanotubes (Figure 1) to better understand how the dipole moments of FF molecules form the helix-like PNT with different chiralities and quantify the chirality.

The dipole moments, **D***_i_*, of L-FF and D-FF molecules and corresponding helix-like structures were calculated using the semi-empirical quantum-mechanical method PM3 in the restricted Hartree-Fock approximation (RHF) and molecular mechanical force field method Amber from the HyperChem package [62]. Previous studies [37,38,42] have shown that for the dipole moments and energy calculated with other methods AM1 and BIOCHARM are similar to those obtained by PM3 and Amber. Therefore, in this work we used only PM3 and Amber methods. The calculated values of the dipole moments for individual L-FF and D-FF molecules are presented in Table 2. They are similar to the results obtained and analyzed earlier [30,37,38,42], and correspond well to the molecules’ orientation in experimentally observed helix-like structures.

## 3. Results and Discussions

In contrast to α-helix proteins, supramolecular PNTs are comprised of individual FF molecules held by relatively weak hydrogen bonds [31,32,33]. Therefore, the chirality quantification method developed earlier for protein structures [21,22,23,24] cannot be directly applied for PNTs and requires some adaptations.

**Figure 4:**
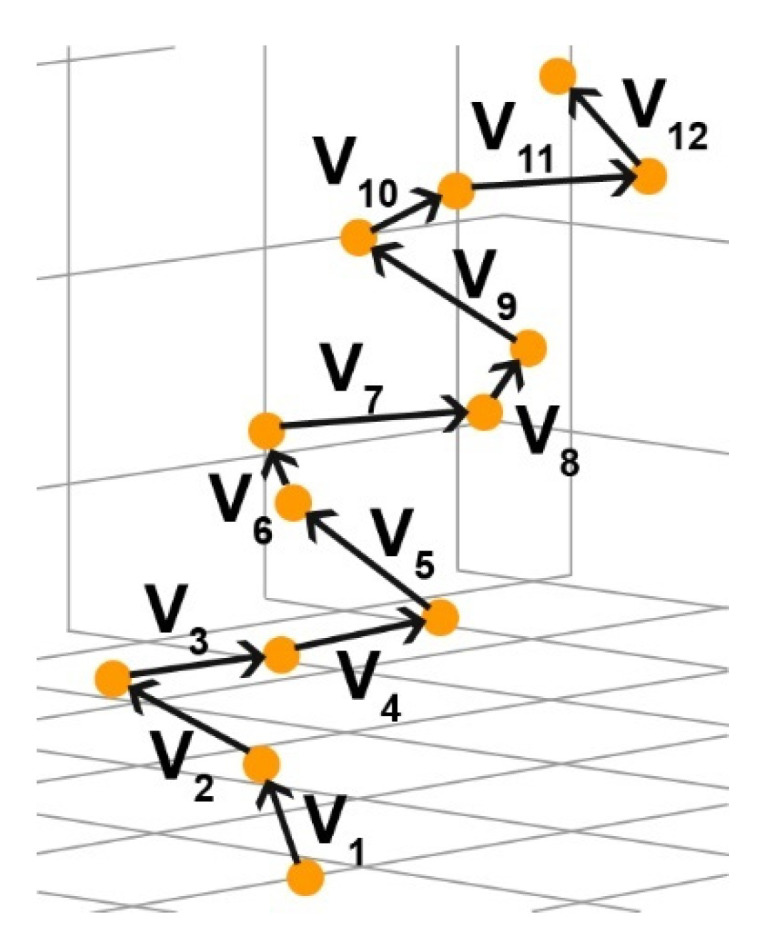
Graphical representation of vectors between neighboring Cα atoms in a helical protein used for calculating a scalar triple product.

Briefly, the original method considered a helical polypeptide chain consisting of *n* amino acid residues, and a set of (*n* − 1) vectors $v_{i}$ was built between each two adjacent Cα atoms in amino acid residues (Figure 4). For each three consecutive vectors, their scalar triple product was calculated:

(1) $$\left(\left[v_{1},v_{2}\right],v_{3}\right)=\left(y_{1}z_{2}-y_{2}z_{1}\right)x_{3}+\left(z_{1}x_{2}-z_{2}x_{1}\right)y_{3}+\left(x_{1}y_{2}-x_{2}y_{1}\right)z_{3},$$

The sum of all these scalar triple products (see Equation (2)) allowed us to estimate the chirality sign. If *χ_total_* is positive, the structure is right-handed; for left-handed structures, *χ_total_* is negative [23,24]. This method has been validated for almost 1000 proteins [23,24] taken from the Protein Data Bank [25].

(2) $$\chi_{total}=\sum_{i=1}^{n-3}\left(\left[v_{i},v_{i+1}\right],v_{i+2}\right)$$

In this work, the abovementioned approach was adopted for analysis of supramolecular FF PNTs. Instead of vectors between adjacent Cα atoms, a scalar triple product of dipole moments **D***_i_* of the successive individual FF molecules constituting a turn of the PNT helix-like nanotube was used. The origin of **D***_i_* vectors is taken relative to the center of mass of the corresponding molecules. The absolute value of each dipole moment **D***_i_* is

(3) $$D_{i}=\left|D_{i}\right|=\sqrt{D_{x,i}^{2}+D_{y,i}^{2}+D_{z,i}^{2}},$$

where *D_x,i_*, *D_y,i_*, and *D_z,i_* are the components of the *i*-th vector **D***_i_* in the Cartesian coordinates. Similar to Equation (2), the sum of the scalar triple products of the dipole moments related to the PNT’s chirality can be written as:

(4) $$c_{total}=\sum_{i=1}^{n-2}\left(\left[D_{i},D_{i+1}\right],D_{i+2}\right).$$

It is necessary to note that the summation here is taken over *i* in the range from 1 to (*n* − 2), whereas in Equation (2), the *i* range is from 1 to (*n* − 3). This is because in supramolecular helixes *i* numerates the individual molecules instead of the Cα atoms in proteins. The *c_total_* can be normalized over the average value of the total dipole momentum of the PNT’s coil, $D_{av}=\frac{1}{6}\overset{6}{\underset{i=1}{\sum }}D_{i}$, to find a universal measure of the chirality:

(5) $$c_{norm}=\frac{c_{total}}{D_{av}^{3}}.$$

**Figure 5:**
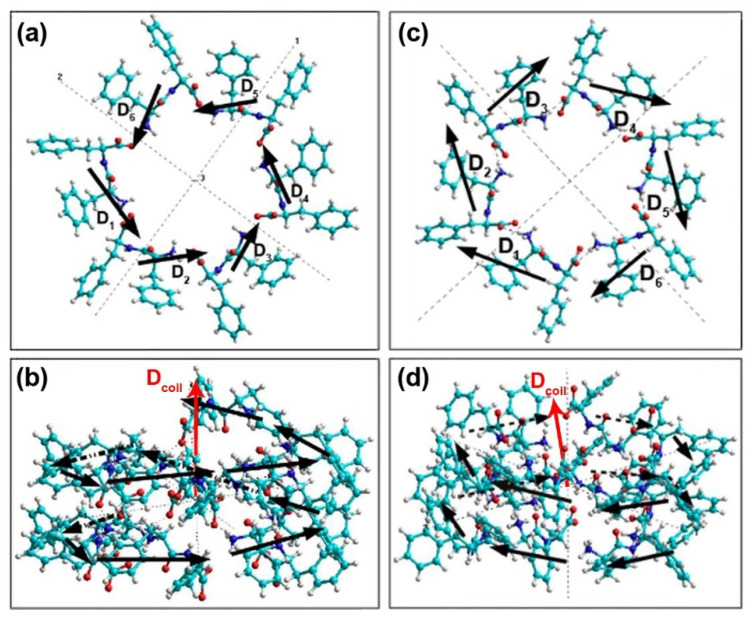
Schematic presentation of dipole moments **D***_i_* in two coils of (**a**,**b**) L-FF and (**c**,**d**) D-FF PNTs: (**a**,**c**) Z-plane projection, (**b**,**d**) Y-plane projection. For L-FF PNT dipole moments form a right-hand helix, whereas for D-FF PNT form a left-hand helix. Red arrows show the directions of the total dipole moments of the coil **D***_coil_*.

Individual dipole moments of FF molecules in one coil of helix-like PNTs were obtained using semiempirical PM3 method in restricted Hartree–Fock (RHF) approximation and molecular mechanic Amber method (after PM3) implemented in HyperChem software [62]. The results are presented below in Table 3 and Table 4 for L-FF and for D-FF, respectively. Schematic representation of the spatial arrangement of FF individual dipole moments **D***_i_* in two coils of PNTs are shown in Figure 5a,b for L-FF and in Figure 5c,d for D-FF PNTs.

It is important to note that, due to the helix-like structure of PNT, the dipole moment **D***_i_* of each next FF molecule in the coil is rotated by ~60° in the XOY plane. Therefore, at a full vector rotation at 360 degrees in a coil, the components *D_i,x_* and *D_i,y_* almost compensate for one another. Thus, the *x* and *y* components of the total dipole moment of the coil, *D_coil_*, are much smaller than *D_coil,z_*, which is always oriented along the OZ axis and increases *D_coil_* (Table 3 and Table 4). As a result, the total dipole moment of a coil *D_coil_* is directed mainly along OZ axis with slight deviations (Figure 5), which corresponds to the previously obtained data [30,37,38,56,57].

It is worth noting that, in contrast to the original chirality quantification method developed for proteins [21,22,23,24], where the vectors were built between the carbon atoms Cα of each subsequent amino acid, in the current modification of the method, the vector of the dipole moment of each FF molecule in the PNT is taken relative to the center of mass of the corresponding molecule. The origin of **D***_i_* is a point in space defined by the vector **r*_Di_*** with components {*x_Di_*; *y_Di_*; *z_Di_*} calculated as follows:

$$x_{Di}=\left\{\sum_{j=1}^{N}m_{j}\cdot x_{j}\right\}/\left\{\sum_{j=1}^{N}m_{j}\right\},$$

$$y_{Di}=\left\{\sum_{j=1}^{N}m_{j}\cdot y_{j}\right\}/\left\{\sum_{j=1}^{N}m_{j}\right\},$$

$$z_{Di}=\left\{\sum_{j=1}^{N}m_{j}\cdot z_{j}\right\}/\left\{\sum_{j=1}^{N}m_{j}\right\}.$$

**Figure 6:**
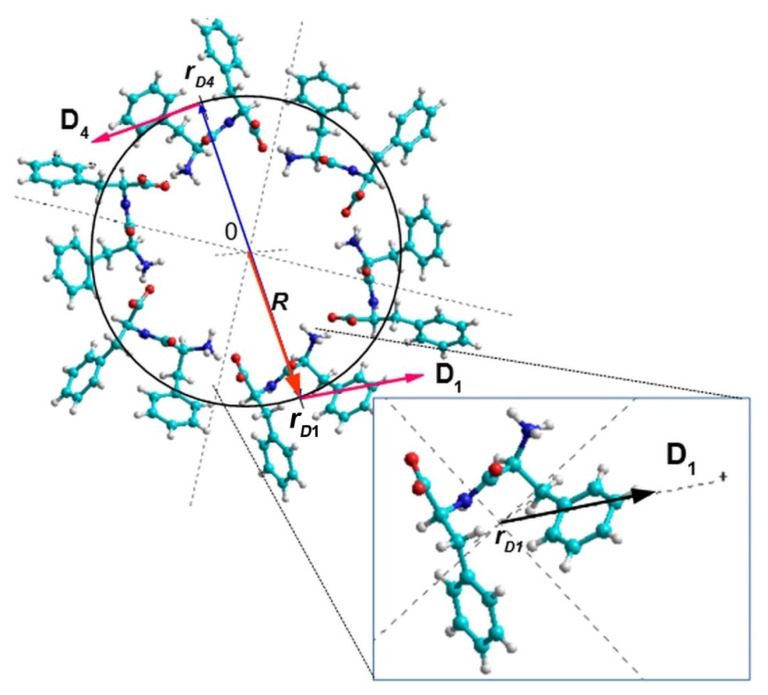
A coil of L-FF PNT with vectors **r**_D1_ and **r**_D4_ pointing the centers of mass of individual FF molecules with dipole moments **D**_1_ and **D**_4_, correspondingly.

Here, *m_j_*, *x_j_*, *y_j_*, and *z_j_* are the mass and coordinates, respectively, of the *j*-th atom in the *i*-th FF molecule in the PNT, and *N* = 43 is the number of atoms in one FF molecule. For example, the coordinates of the center of mass for the first FF molecule (*i* = 1) in a coil of L-FF PNT (Figure 6) are:

*x_D_*_1_ = 2.35 Å; *y_D_*_1_ = −7.80 Å; *z_D_*_1_ = 1.02 Å.

This point is the origin for the **D**_1_ dipole moment vector. Similarly, the origins for other vectors **D***_i_* can be calculated. As a result, dipole moments form a helix with a pitch equal to *c* = 5.456 Å (for L-FF, *c* = 5.441 Å for D-FF PNTs), and the helix radius *R* is about 8.15 Å for L-FF PNT.

The obtained values of the dipole moments for L-FF and D-FF PNTs allow us to quantify their chiralities following the Equations (4) and (5). The calculated magnitudes of the PNTs chirality, *c_total_*, and the normalized chirality, *c_norm_*, are presented in Table 5. For each type of PNT, both PM3 and Amber methods *c_total_* demonstrate close values with the divergence about 15%, whereas for *c_norm_* the divergence is less than 5%. Therefore, *c_norm_* can be considered as a universal value for chirality quantification that does not depend on the calculation method.

At the same time, the absolute value of *c_norm_* for L-FF PNT is about 10% higher than that for D-FF PNT. This difference exceeds the calculation error and thus shows the difference in PNTs chiralities. Lower *c_norm_* value observed for D-FF PNTs indicates that this PNT is twisted tighter than L-FF. This is also confirmed by the lower volume of the D-FF PNT unit cell (Table 1).

Following the original method [23,24], the sign of the *c_norm_* corresponds to the PNT’s chirality type. For L-FF PNT, *c_norm_* is positive thus this PNT should be right-handed, whereas negative *c_norm_* value for D-FF PNT indicates its left-handed twisting. This result is confirmed by the previous crystallographic studies [31,38] and the individual dipole moments completely follow this arrangement (Figure 5). It is worth noting that the chirality alternating observed earlier for natural proteins and DNA [9,10,11,12] preserves in the supramolecular PNTs as well. The type of chirality of the helix-like PNT is opposite to that of the individual dipeptide. This fact also can be a confirmation of the adequacy of the proposed method for supramolecular PNTs chirality quantification.

## 4. Conclusions

A method for quantification of the chirality of self-assembled helix-like FF nanotubes based on the scalar triple products of the individual FF molecules dipole moments is described. The dipole moments were calculated for nanotubes comprised of L-FF and D-FF molecules by quantum-chemical and molecular mechanics methods, and the independence of the magnitude and the sign of the chirality on the calculation method is demonstrated. The obtained magnitudes of the chirality for L-FF nanotubes are about 10% higher than those of L-FF, which indicates that D-FF nanotubes are twisted tighter than L-FF. The alternating of the chirality type observed earlier for natural proteins and DNA also preserves in the supramolecular PNTs. The type of chirality of the helix-like PNT is opposite to that of the individual dipeptide. This effect is in line with the chirality alternation rule, general for different levels of hierarchical organization of molecular systems, and additionally corroborates the validity of the proposed method.

The extension of the chirality quantification method to supramolecular helix-like nanostructures opens new facilities for comparing both molecular constructs of the same chirality type and those with different constructs. Moreover, it provides an opportunity to reveal the physical basis for the chirality sign formation, which is associated with the electrostatic dipole–dipole interaction of individual molecules. This approach can be applied to study other helical and helix-like supramolecular structures.

## Figures and Tables

**Table 1:** Experimental lattice cell parameters for L-FF [31] and D-FF [38] PNTs and inner cavity sizes *R*_1_, *R*_2_ [56].

	**L-FF**	**D-FF**
Space group	P6_1_	P6_5_
*a*, Å	24.0709	23.9468
*b*, Å	24.0709	23.9468
*c*, Å	5.4560	5.4411
*V*, Å^3^	2737.7	2702.2
*R*_1_, Å	15.3	15.2
*R*_2_, Å	12.2	12.1

**Table 2:** The dipole moments and some other structural parameters of individual L-FF and D-FF molecules calculated by PM3 method.

Molecule	Dx, Debye	Dy, Debye	Dz, Debye	Dtot, Debye	Van der Waals Volume, Å^3^	Polarization, C/m^2^	Total Energy, a.u.	RMS Gradient, a.u./Å
L-FF	11.645	1.115	0.899	11.733	291.919	0.134	−133.959	~0.06
D-FF	−11.630	1.052	1.113	11.730	291.977	0.134	−133.959	~0.07

**Table 3:** Values of dipole moments for a coil of the helix-like L-FF PNT computed using PM3 (RHF) and Amber (after PM3) methods. All values of dipole moments are given in Debye units.

*i*	PM3 RHF				Amber			
	*Di*	*Dx*	*Dy*	*Dz*	*Di*	*Dx*	*Dy*	*Dz*
1	24.022	14.576	−15.421	−11.261	23.458	14.901	−15.250	−9.781
2	22.549	−6.313	−18.923	−10.513	21.734	−6.280	−18.879	−8.748
3	22.389	−18.646	−3.636	−11.849	21.545	−18.698	−3.629	−10.070
4	22.381	−11.564	14.461	−12.573	21.530	−11.695	14.495	−10.801
5	22.441	7.555	17.308	−12.123	21.578	7.397	17.408	−10.384
6	22.587	18.767	2.568	−12.303	21.638	18.581	2.745	−10.742
*Dcoil*	70.851	4.376	−3.643	−70.622	60.752	4.206	−3.109	−60.526
*Dav*	22.728	0.729	−0.607	−11.770	21.914	0.701	−0.518	−10.088

**Table 4:** Values of dipole moments for a coil of the helix-like D-FF PNT computed using PM3 (RHF) and Amber (after PM3) methods. All values of dipole moments are given in Debye units.

*i*	PM3 RHF				Amber			
	*Di*	*Dx*	*Dy*	*Dz*	*Di*	*Dx*	*Dy*	*Dz*
1	22.523	−12.228	−15.267	−11.167	21.707	−12.299	−15.170	−9.475
2	22.340	7.302	−18.014	−11.072	21.527	7.210	−17.995	−9.360
3	22.372	19.234	−2.597	−11.125	21.520	19.168	−2.656	−9.416
4	22.475	11.905	15.905	−11.290	21.625	11.914	15.274	−9.612
5	22.629	−6.613	17.478	−12.761	21.703	−6.487	17.386	−11.256
6	23.855	−19.820	4.382	−12.531	23.271	−19.893	4.727	−11.112
*Dcoil*	69.971	−0.218	1.888	−69.945	60.253	−0.387	1.565	−60.231
*Dav*	22.704	−0.036	0.315	−11.658	21.892	−0.064	0.261	−10.038

**Table 5:** Magnitudes and signs of the chirality obtained for L-FF and D-FF PNTs for various calculating methods.

Type of PNT	L-FF		D-FF	
Calculating Method	PM3	Amber	PM3	Amber
***c***_total_, Debye^3^	16,034.82	13,870.71	−14,497.03	−12,161.23
***c***_norm_	1.37	1.32	−1.23	−1.16
Chirality sign	positive	positive	negative	negative
Chirality symbol	D	D	L	L

## Data Availability

The data presented in this study are available on request from the corresponding author.
